# Hints to Practitioners

**Published:** 1898-01-29

**Authors:** 


### HINTS TO PRACTITIONERS.

### The Examination of the Teeth in Phthisis.

Dr. Graham Crookshank, in a paper read before the Medical Society of University College, says that it is of the greatest importance to examine the teeth in all cases of phthisis, real or suspected. Many cases of indigestion depending on carious teeth have been diagnosed as early phthisis, the suppuration having been free enough to set up mild symptoms of septic absorption, and the mimicry completed by lienteric diarrhœa due to the imperfectly masticated food. But even where there is no doubt about the phthisis no effort should be spared to have the teeth put in good order, so as to regain the power of proper mastication.

